# The Heme Oxygenase System Rescues Hepatic Deterioration in the Condition of Obesity Co-Morbid with Type-2 Diabetes

**DOI:** 10.1371/journal.pone.0079270

**Published:** 2013-11-15

**Authors:** Tatiana Ntube Salley, Manish Mishra, Shuchita Tiwari, Ashok Jadhav, Joseph Fomusi Ndisang

**Affiliations:** Department of Physiology, University of Saskatchewan College of Medicine, Saskatoon, Saskatchewan, Canada; Université Libre de Bruxelles, Belgium

## Abstract

The prevalence of non-alcoholic fatty-liver disease (NAFLD) is increasing globally. NAFLD is a spectrum of related liver diseases that progressive from simple steatosis to serious complications like cirrhosis. The major pathophysiological driving of NAFLD includes elevated hepatic adiposity, increased hepatic triglycerides/cholesterol, excessive hepatic inflammation, and hepatocyte ballooning injury is a common histo-pathological denominator. Although heme-oxygenase (HO) is cytoprotective, its effects on hepatocyte ballooning injury have not been reported. We investigated the effects of upregulating HO with hemin or inhibiting it with stannous-mesoporphyrin (SnMP) on hepatocyte ballooning injury, hepatic adiposity and inflammation in Zucker-diabetic-fatty rats (ZDFs), an obese type-2-diabetic model. Hemin administration to ZDFs abated hepatic/plasma triglycerides and cholesterol, and suppressed several pro-inflammatory cytokines and chemokines including, TNF-α, IL-6, IL-1β, macrophage-inflammatory-protein-1α (MIP-1α) and macrophage-chemoattractant-protein-1 (MCP-1), with corresponding reduction of the pro-inflammatory M1-phenotype marker, ED1 and hepatic macrophage infiltration. Correspondingly, hemin concomitantly potentiated the protein expression of several markers of the anti-inflammatory macrophage-M2-phenotype including ED2, IL-10 and CD-206, alongside components of the HO-system including HO-1, HO-activity and cGMP, whereas the HO-inhibitor, SnMP abolished the effects. Furthermore, hemin attenuated liver histo-pathological lesions like hepatocyte ballooning injury and fibrosis, and reduced extracellular-matrix/profibrotic proteins implicated in liver injury such as osteopontin, TGF-β1, fibronectin and collagen-IV. We conclude that hemin restore hepatic morphology by abating hepatic adiposity, suppressing macrophage infiltration, inflammation and fibrosis. The selective enhancement of anti-inflammatory macrophage-M2-phenotype with parallel reduction of pro-inflammatory macrophage-M1-phenotype and related chemokines/cytokines like TNF-α, IL-6, IL-1β, MIP-1α and MCP-1 are among the multifaceted mechanisms by which hemin restore hepatic morphology.

## Introduction

Obesity is associated with many health complications including, type-2 diabetes, hyperlipidemia, dyslipidemia, hypertension and non-alcoholic fatty liver disease (NAFLD) [1]–[6]. NAFLD is a wide spectrum of related liver diseases that progressive from simple a condition like steatosis to a more serious complication like cirrhosis, and elevated hepatic adiposity, high levels of hepatic triglycerides and hepatic cholesterol and hepatocyte ballooning injury are common denominators of NAFLD [1]–[5], [7]–[9]. Besides hepatic adiposity, inflammation is crucial in the pathogenesis of NAFLD. Elevated levels of pro-inflammatory cytokines such as tumor necrosis factor (TNF-α), interleukin (IL)-6, IL1β are amongst the pathophysiological driving force of NAFLD [10], [11]. Similarly pro-inflammatory chemokines such as macrophage inflammatory protein-1 alpha (MIP-1α) and macrophage chemoattractant-protein-1 (MCP-1) are known to trigger macrophage infiltration to accentuate hepatic inflammatory insults [12], [13] and compromise hepatic morphology and function. Generally, two common forms of macrophages have been described [14], [15]. These include the pro-inflammatory macrophage M1-phenotype that is stimulated by cytokines and chemokines like TNF-α, IL-6, IL-1β, MIP-1α and MCP-1 [16], [17], and the anti-inflammatory M2-phenotype that is associated with IL-10 [18]–[20]. Therefore substances capable of selectively modulating the polarization of macrophages towards the anti-inflammatory M2-phenotype and concomitantly reducing the pro-inflammatory M1-phenotype and its related secretagogues like TNF-α, IL-6, IL-1β, MIP-1α, MCP-1 and abates excessive hepatic triglycerides and hepatic cholesterol may suppress and/or retard the progression of NAFLD to more severe conditions like hepatic cirrhosis.

Fatty liver contributes significantly to obesity-related morbidity and mortality [21]–[23]. Pharmacological agents that can rescue the liver from lipotoxicity by restoring adipose tissue insulin sensitivity or preventing activation of inflammatory and oxidative insults hold promise in the treatment and management of NAFLD, although their long-term safety and efficacy remains to be clearly established [24]. The soaring prevalence of NAFLD necessitates the development of new therapeutic modalities to improve and possibly reverse the clinical symptoms of NAFLD. An interesting physiological enzyme that could be explored in this regard is heme-oxygenase (HO). HO is a microsomal enzyme with two active isoforms HO-1 (inducible) and HO-2 (constitutive), while the third isoform, HO-3 is a pseudo-transcript of HO-2 without catalytic activity [14]. The HO-system can be pharmacologically enhanced to modulate physiological functions and combat adversity in tissue [14], [25]–[27]. Although emerging evidence indicates that an upregulated HO-system is capable of suppressing visceral adiposity [28], [29], however, the effect of the HO-system on hepatic adiposity has not been reported. More-importantly, the role of the HO system on hepatocyte ballooning injury has not been reported. Similarly, the pathophysiology of hepatocyte ballooning injury in Zucker diabetic fatty rats (ZDFs), a model characterized by obesity and type-2 diabetes, with aberrant hepatic response to insulin [30] and impaired hepatic lipid metabolism [31] remains largely unclear. Furthermore, the role of the HO-system on macrophage M1/M2-phenotype in hepatic tissue from ZDFs has not been reported. Understanding the pathophysiological perturbations that accompanies hepatic impairment would have important implications for the prevention and treatment of diseases associated with fatty liver.

Therefore this study will examine the effects of the HO-system on the pathophysiology of hepatocyte ballooning, macrophage M1/M2-phenotypes and related cytokines and chemokines such as TNF-α, IL-6, IL-1β, MIP-1α and MCP-1, IL10 in the liver of ZDFs. In addition, the effects of hemin therapy on extracellular-matrix/profibrotic proteins implicated in hepatic injury like osteopontin, transforming growth factor-beta (TGF-β1), fibronectin and collagen-IV [32]–[36] were examined.

## Materials and Methods

### Animals and Treatment Groups

Our experimental protocol was approved by the Animal Care and Research Ethics Committee of University of Saskatchewan, which is in conformity with the Guide for Care and Use of Laboratory Animals by the Canadian Council on Animal Care and the National Institutes of Health (NIH Publication No. 85-23, revised 1996). Male ZDFs of twelve weeks and age/sex-matched Zucker-lean (ZL) littermates were bought from Charles River (Willington, MA, USA), and housed at 21°C with 12-hour light/dark cycles, fed with Purina 5008 diet and had access to drinking water *ad libitum*. The animals were allowed to accommodate for two weeks. At 14 weeks of age, the animals were randomly assigned to the following experimental groups (*n = 6* per group): **(A)** controls (ZDF and ZL), **(B)** hemin-treated ZDF and ZL, **(C)** ZDF+hemin+SnMP, **(D)** ZDF+SnMP, and **(E)** ZDF+vehicle dissolving hemin and SnMP. The HO-inducer, hemin (Sigma, St Louis, MO) was administered twice weekly for a duration of eight weeks at a dose of 30 mg/kg intraperitoneally, while the HO-inhibitor, stannous-mesoporphyrin (SnMP, Porphyrin Products, Logan, UT) was given at a dose of (2 mg/100 g body weight) by intraperitoneal injection twice weekly for 8 weeks as we previously reported [29]. During the treatment period body weight and glucose levels were determined on a weekly routine after 6 hrs of fasting in metabolic cages using glucose-meter (BD, Franklin Lakes, NJ, USA). The experiments were terminated at the end of the 8-week treatment period, and the age of the animals was 22 weeks. Before killing, the animals were weighed and anaesthetized with pentobarbital sodium (50 mg/kg i.p.), and when the animals were fully unconscious blood was obtained by cardiac puncture. Subsequently, an incision was made in the peritoneum and the liver was carefully isolated, cleaned in ice-cold phosphate-buffered saline and weighed using an analytical balance (Precisa Instruments Ltd, Switzerland) as previously reported [37]–[39]. The liver and plasma were used for biochemical assays.

### Histological, Morphological and Immunohistochemical Analyses of Kidney Tissue

Histological and morphometric studies were done as we previously reported [28]. Liver sections of 5 µm were cut and treated with Masson’s trichrome staining to assess collagen deposition and hepatocyte ballooning injury using a microscope (Aperio Scan Scope, Model CS, Aperio Technology Inc, CA). Morphologic assessment of hepatocyte ballooning injury was done by a blinded researcher using a microscope (Aperio Scan Scope, Model CS, Aperio Technology Inc, CA), and analyzed using Aperio Image Scope V11.2.0.780 software (Aperio, e-Pathology Solution, CA). Liver sections were magnified at 200X, and 20 random snaps shots were taken per slide for each experimental group of 4–6 animals (80–120 images per group). The images were scored semi-quantitatively by a blinded researcher as we previously reported [28], [29].

Immunohistochemistry was done as we previously reported [40]. Sections of 5 µm of whole liver sections were treated with bovine serum albumin in phosphate buffered saline to block non-specific staining and incubated overnight with ED1 antibody (1∶500 dilution, Santa Cruz Biotechnology, CA). Subsequently, the liver sections were incubated with goat anti-mouse IgG for 30 minutes (1∶200 dilution; Jackson ImmunoResearch Laboratories, Inc., ME, USA). Immunohistochemical staining was done using the standard avidin-biotin complex method with the chromagen 3,3′-diaminobenzidine (DAB) at the final detection step. The liver sections were scanned using a virtual microscope (Aperio Scan Scope, Model CS, Aperio Technology Inc, CA). Macrophages (brown from immune-stained sections) were quantified by a blinded researcher by manually counting the positively-stained ED1 cells under 200 X magnification in 15 randomized non-overlapping fields, and only distinct ED1-stained cells from all experimental groups were taken into consideration.

### Determination of HO Activity and HO-1 Concentration

Liver HO activity was measured as bilirubin production using our established method [41]–[43]. The amount of bilirubin in each sample was determined spectrophotometrically (extinction coefficient for bilirubin 40 mM^−1^cm^−1^), and expressed as nmole/mg protein/hour.

Hepatic HO-1 concentration was determined using enzyme-linked immunosorbent assay (ELISA, Stressgen-Assay Design, Ann Arbor, MI, USA) as we previously reported [41]–[43].

### Measurement of TNF-α, IL-6 and IL-1β

The levels of TNF-α, IL-6 and IL-1β in the liver was assessed by using ELISA kits (Immuno-Biological Laboratories Co Ltd, Takasaki-shi, Gunma, Japan) following to the manufacturer’s instructions and read at 450 nm in a plate reader (SpectraMax 340PC, Molecular Device, CA, USA) as we previously reported [29].

### Western Immunoblotting

The liver was homogenized (1∶10, w:v) in 10 mM Tris-buffered saline (20 mM Tris-HCl, pH 7.4, 0.25 M sucrose, and 1 mM EDTA) in the presence of a cocktail of protease inhibitors, centrifuged, and proteins extracted and quantified as we previously reported [44], [45]. Aliquots of 50 µg of proteins were loaded on SDS-polyacrylamide gel, and the fractionated proteins were electrophoretically transferred to nitrocellulose paper. Non-specific bindings was blocked with 3% non-fat milk, and incubated overnight with primary antibodies against ED1, ED2, CD206, IL-10, TGF-β1/2/3, collagen-IV, fibronectin and osteopontin (Santa Cruz Biotechnology, CA, USA). Anti-mouse Beta-actin (Sigma St Louis, MO, USA) was used as control to ascertain equivalent loading. After washing, blots were incubated with anti-rabbit IgG conjugated to horseradish peroxide (Bio-Rad, CA, USA), and the immuno-reactivity visualized using enhanced horseradish peroxide/luminol chemiluminescence reagent (Perkin Elmer Life Sciences, Boston, MA, USA). Densitometric analysis was done with UN-SCAN-IT software (Silk Scientific, Utah, USA).

### Determination of cGMP

The concentration of cGMP in the liver determined using an enzyme-immunoassay kit (Cayman Chemical, Ann Arbor, MI, USA) as previously described [41], [42]. Briefly, homogenized liver tissue was treated with 6% trichloroacetic acid at 4°C in the presence of 3′-isobutyl-1-methylxanthine to inhibit phosphodiesterase activity. The samples were subsequently centrifuged at 2000 *g* for 15 minutes and the supernatant was recovered, washed with water-saturated diethyl ether and the upper ether layer aspired and discarded while the aqueous layer containing cGMP was recovered and lyophilized. The dry extract was dissolved in 1-ml assay buffer and the cGMP was assessed according to the manufacturer’s instruction and expressed as picomol per mg of protein.

### Determination of MCP-1 and MIP-1α

The concentrations of macrophage inflammatory protein-1 alpha (MIP-1α) and macrophage chemoattractant protein-1 (MCP-1) in the liver were determined using ELISA kits (OmniKine™, Assay Biotechnology Company Inc, Sunnyvale, CA). All samples were assayed in triplicates following the manufacturer’s instructions.

### Determination of Triglycerides and Total Cholesterol

Total-cholesterol and triglycerides were measured in liver homogenates and plasma using cholesterol and triglycerides assay kits (Cayman Chemical, Ann Arbor, MI, USA) following instructions from the manufacturer.

### Statistical Analysis

All data are expressed as means ± SEM from at least four independent experiments unless otherwise stated. Statistical analyses were done using two-way ANOVA, by means of Statistical Analysis System (SAS), software, version 9.3 (SAS Institute Inc., Cary, NC, USA) and Student’s *t-*test. Group differences at the level of p<0.05 were considered statistically significant.

## Results

### Hemin Therapy Potentiates the HO-system, Normalized Glycaemia and Reduced Liver Hypertrophy

The administration of the HO-inducer, hemin, to ZDFs normalized glycaemia (26.3±2.5 *vs* 8.3±1.4 mmol/L, p<0.01) and reduced liver-to-body weight ratio, an important index of liver hypertrophy [46] (54.8±1.8 *vs* 40.7±1.5 g/Kg body weight, p<0.01) (**Table 1**). In contrast, co-administrating hemin and the HO-inhibitor, SnMP abolished the effect of hemin on liver hypertrophy (54.8±1.8 *vs* 55.2±2.3 g/Kg body weight) and glycaemia (26.3±2.5 *vs* 29.4±3.7 mmol/L). On the other hand, treatment with hemin together with SnMP resulted in a slight reduction of body-weight, which was less than 10% (**Table 1**). The body-weight loss may not be due to toxicity because we recently reported that important indices of toxicity such as gamma-glutamyltransferase, plasma alanine aminotransferase and aspartate aminotransferase were within normal range [42].

**Table 1:** Effect of hemin and stannous mesoporphyrin (SnMP) on physiological parameters in ZDF and ZL rats.

Physiological parameters	Animal groups					
	Control ZL	ZL+ Hemin	Control ZDF	ZDF+Hemin	ZDF+Hemin +SnMP	ZDF+Vehicle
Body weight (g)	348.6±5.9	341.2±10.5	406.3±5.2†	382.5±4.8*	369.2±5.5#	396.5±9.3
Fasting glucose (mmol/L)	6.5±0.2	5.9±0.3*	26.3±2.5††	8.3±1.4**	29.4±3.7#	25.7±2.6
Liver weight (g/kg body weight)	45.4±1.4	42.2±4.6	54.8±1.8†	40.7±1.5*	55.2±2.3#	53.6±2.8

* p<0.05,

** p<0.01 *vs* Control ZDF or control ZL;

# p<0.05 *vs* ZDF+Hemin;

† p<0.05,

†† p<0.01 *vs* control ZL; n = 6 per group.

Although body-weight loss may affect glucose levels, it is unlikely in this situation because the slight loss of body-weight in hemin-treated ZDFs and hemin+SnMP-treated ZDFs were accompanied by opposing glycemic effects (**Table 1**). Accordingly, in hemin-treated ZDFs there was reduction of hyperglycemia, whereas co-administering hemin and the HO-blocker, SnMP abolished the effects of hemin on glycaemia, suggesting that the HO system may have an intrinsic anti-diabetic effect.

**Figure 1:**
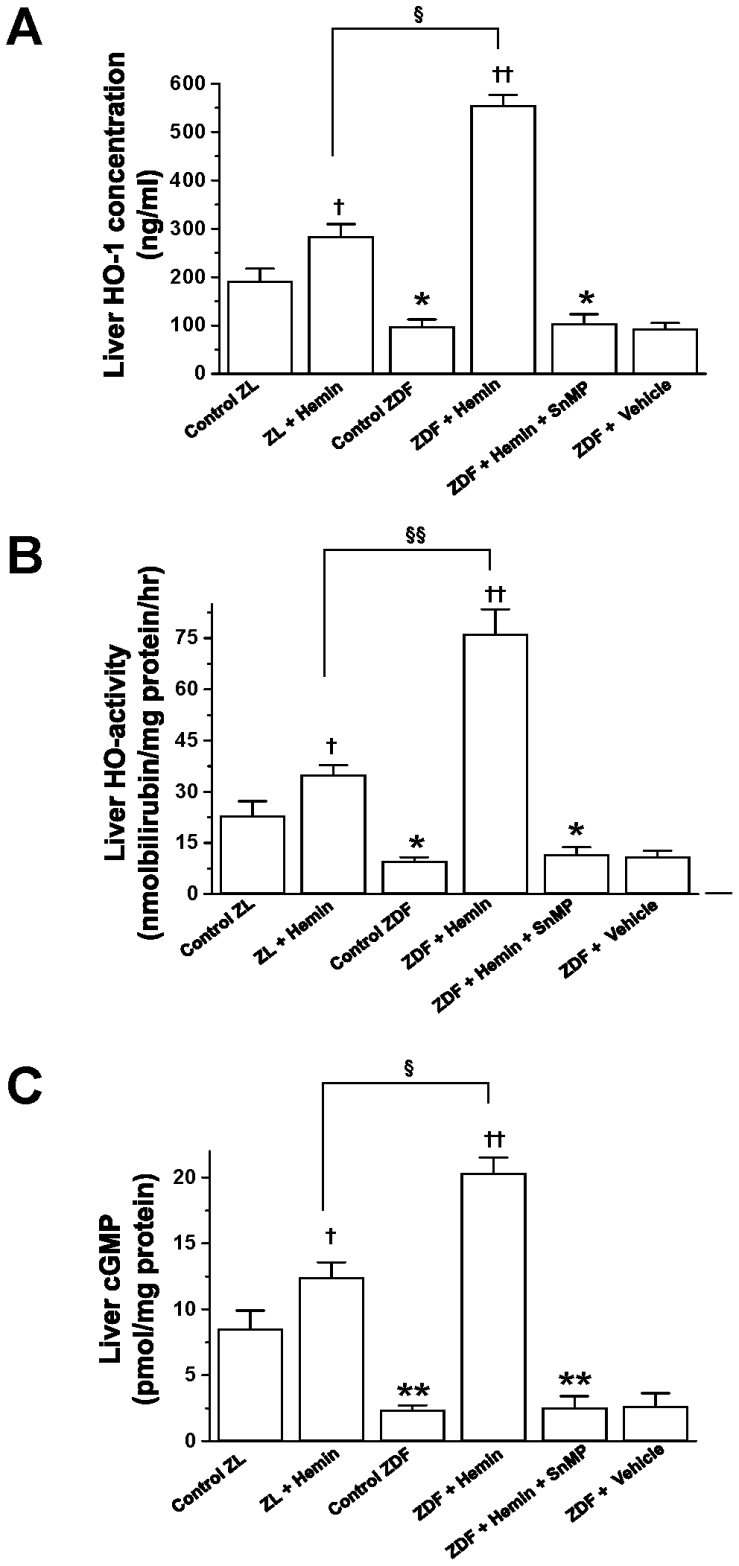
Effects of the HO-inducer, hemin and the HO inhibitor SnMP, on hepatic levels of HO-1, HO activity and cGMP. Hemin therapy greatly increased the depressed (A) HO-1 concentration, (B) HO-activity, and (C) cGMP levels in ZDFs, whereas the HO blocker, SnMP nullified the effects. Treatment of hemin to ZL also resulted to increased levels of HO-1, HO-activity and cGMP in ZLs, thought less effectively as compared to ZDFs. Bars represent means ± SEM; *n = 6* rats per group (*p<0.05, **p<0.01 *vs* Control-ZL; ^†^p<0.05, ^††^p<0.01 *vs* Control-ZL or Control-ZDF; ^§^p<0.05, ^§§^p<0.01 *vs* ZL+Hemin).

To further investigate the role of the HO system on hyperglycemia and liver hypertrophy, we measured important components of the HO system such as HO-1 and HO-activity in the liver. Our results indicate that in ZDF-control, the basal HO-1 and HO-activity were significantly depressed as compared to the ZL-controls (**Figs. 1A and 1B**). Interestingly, hemin administration greatly enhanced the aberrant HO-1 and HO activity in ZDFs, whereas the co-administration of hemin together with the HO inhibitor, SnMP nullified the effect of hemin.

Since enhanced HO activity is accompanied by increased production of endogenous of carbon monoxide that would in turn enhance cGMP [42], we measured cGMP. Moreover, both cGMP and carbon monoxide are known to enhance glucose metabolism [47], [48]. In ZDF-controls, the basal levels of cGMP were markedly reduced as compared to ZL-controls (**Fig. 1C**). However, treatment with hemin robustly enhanced cGMP in ZDFs (**Fig. 1C**), suggesting a role of the HO-cGMP axis in the normalization of hyperglycemia in hemin-treated ZDFs (**Table 1**). In contrast, co-administering hemin with SnMP reversed the effects of hemin.

Hemin therapy also enhanced HO-1, HO-activity and cGMP levels in ZL-controls, although the magnitude was smaller as compared to ZDFs, suggesting greater selectivity of hemin in diseased condition. The vehicle dissolving hemin and SnMP had no effect on any of the measured parameters.

### Hemin Therapy Abated the Elevated Basal Levels of Inflammatory Cytokines in the Liver of ZDFs

**Figure 2:**
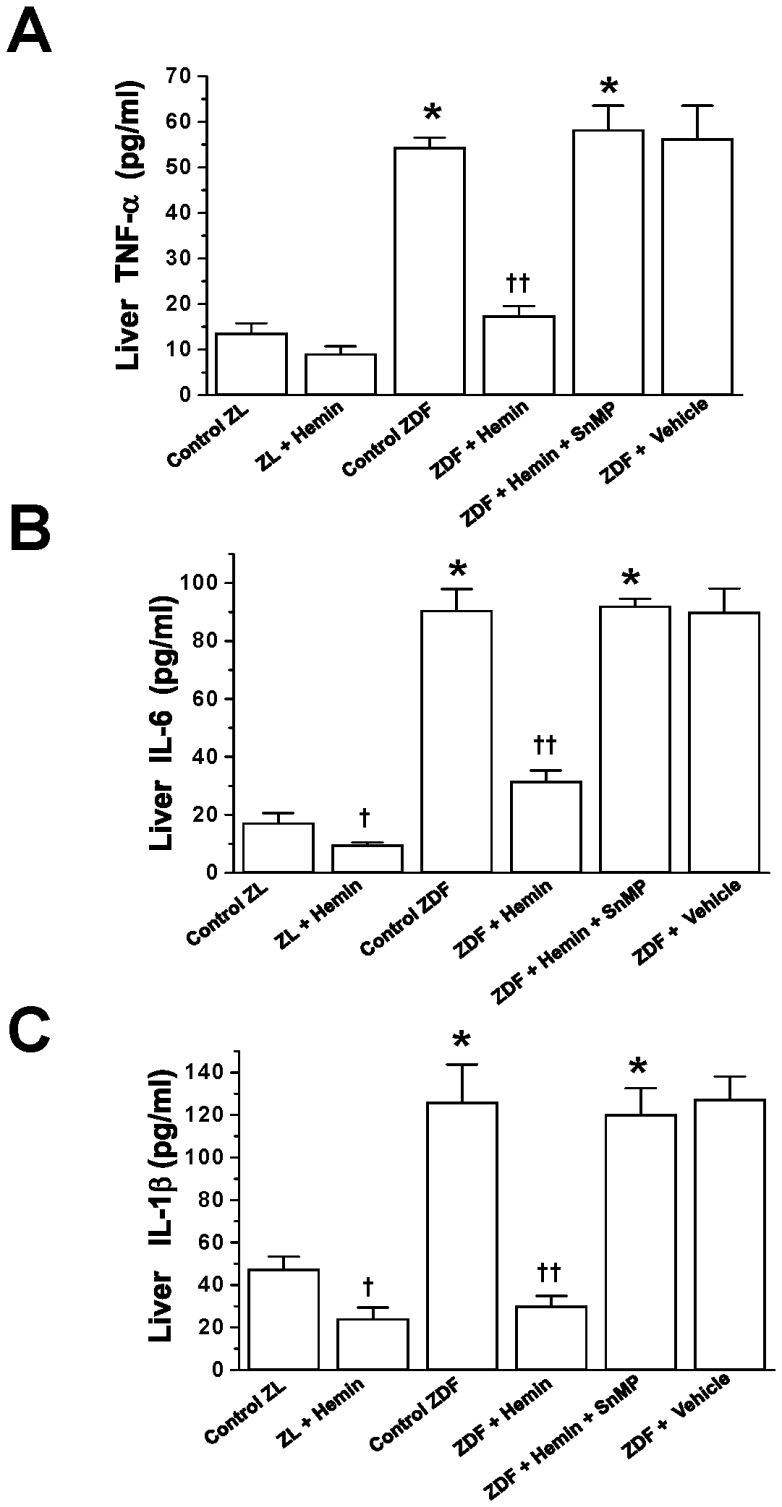
Effects of hemin, the HO inducer and SnMP, the HO inhibitor on liver levels of TNF-α, IL-6 and IL-1β. Hemin therapy attenuated the elevated basal levels of (A) TNF-α, (B) IL-6 and (C) IL-1β in ZDFs, while the HO blocker, SnMP reversed the effects. Hemin therapy also lowered TNF-α, IL-6 and IL-1β levels in ZLs. Bars represent means ± SEM; *n = 6* rats per group (*p<0.01 *vs* Control-ZL; ^†^p<0.05, ^††^p<0.01 *vs* Control-ZL or Control-ZDF).

Given that elevated inflammation due to TNF-α, IL-6 and IL-1β are amongst the causative factors of liver fibrosis and liver steatosis [10], and high levels of TNF-α, IL-6 and IL-1β are known to deregulate glucose metabolism [14], [49], we measured the levels of these cytokines in the liver. Our results indicate that the basal levels of TNF-α, IL-6 and IL-1β in ZDF-controls were significantly elevated as compared to the ZL-controls (**Figs. 2A, 2B and 2C**). Interestingly the normalization of glycaemia in hemin-treated ZDFs was accompanied by the attenuation of TNF-α, IL-6 and IL-1β, whereas co-treatment of hemin and the HO-inhibitor, SnMP, reversed the effects of hemin (**Figs. 2A, 2B and 2C**). Hemin therapy also attenuated TNF-α, IL-6 and IL-1β in the ZL-controls, although to a lesser extent as compared to ZDFs.

### Treatment with Hemin Abated Inflammatory Chemokines such as MCP-1 and MIP-1α in Hepatic Tissue of ZDFs

**Figure 3:**
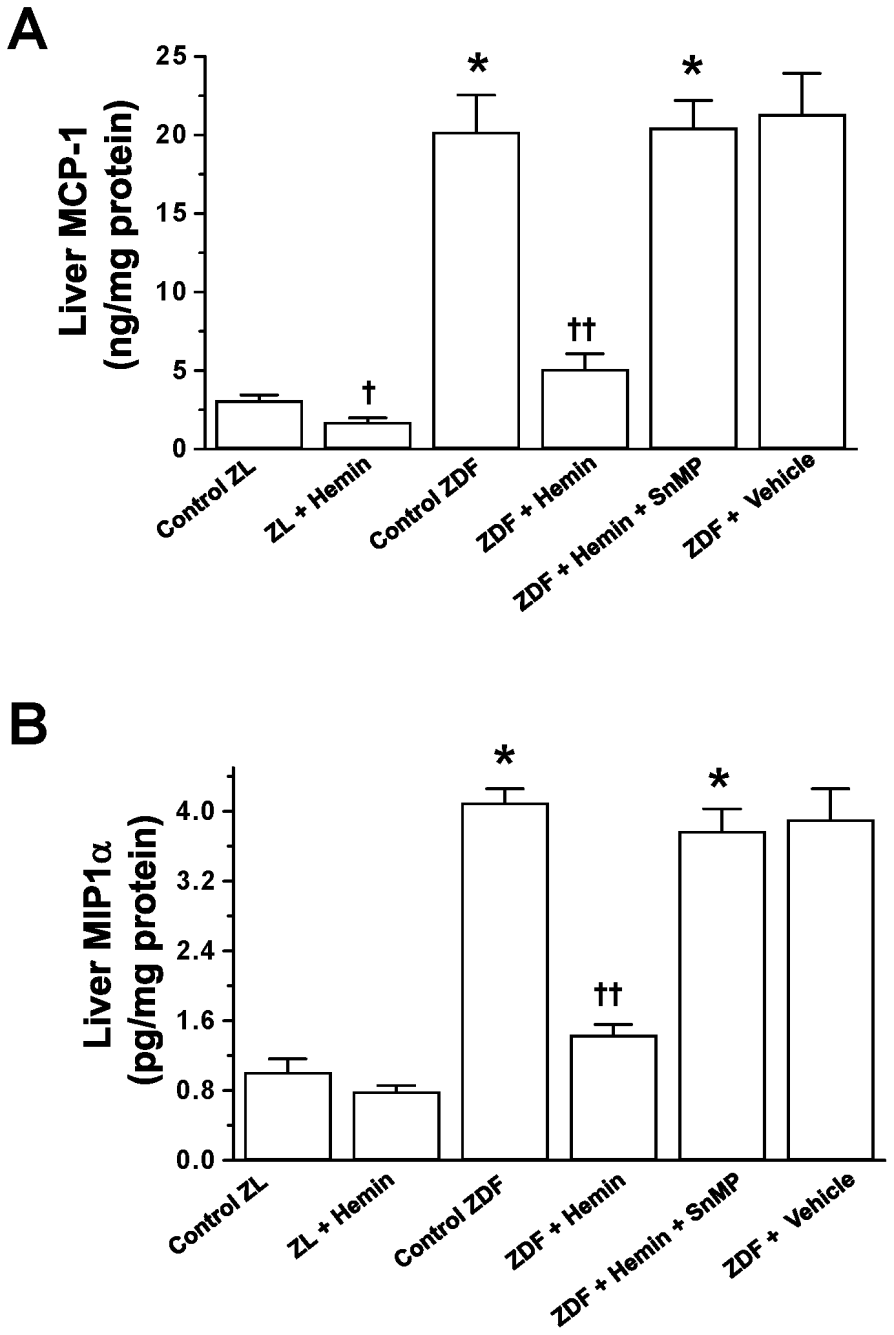
Effects of hemin, the HO inducer and SnMP, the HO inhibitor on hepatic levels of MCP-1 and MIP1-α. Hemin therapy suppressed the elevated basal levels of (A) MCP-1 and (B) MIP1-α in ZDFs, but the HO blocker, SnMP abolished the effects of hemin. Treatment with hemin also reduced MCP-1 and MIP1-α in ZLs. Bars represent means ± SEM; *n = 6* rats per group (*p<0.01 *vs* Control-ZL; ^†^p<0.05, ^††^p<0.01 *vs* Control-ZL or Control-ZDF).

To further investigate the effects of hemin therapy on liver inflammation and hepatic lesions, we measured MIP-1α and MCP-1 since these chomokines trigger macrophage infiltration and cause hepatic injury [12], [13]. In ZDF-controls, the basal levels of MCP-1 levels were markedly elevated as compared to ZL-controls (**Fig. 3A**), but were abated by hemin, whereas the co-treatment of hemin with SnMP nullified the effects of hemin (**Fig. 3A**). Hemin therapy was also effective against MIP-1α in ZDFs (**Fig. 3B**). In ZDF-controls, the basal levels of MIP-1α were significantly enhanced as compared to ZL-controls but were reduced by hemin, whereas the co-administration of hemin with SnMP abolished the effects of hemin (**Fig. 3B**).

Hemin therapy also reduced MCP-1 and MIP-1α in ZL-controls, although the effect in hemi-treated ZLs was smaller as compared to hemin-treated ZDFs.

### Hemin Therapy Abated Plasma and Hepatic Triglycerides and Cholesterol in ZDFs

**Figure 4:**
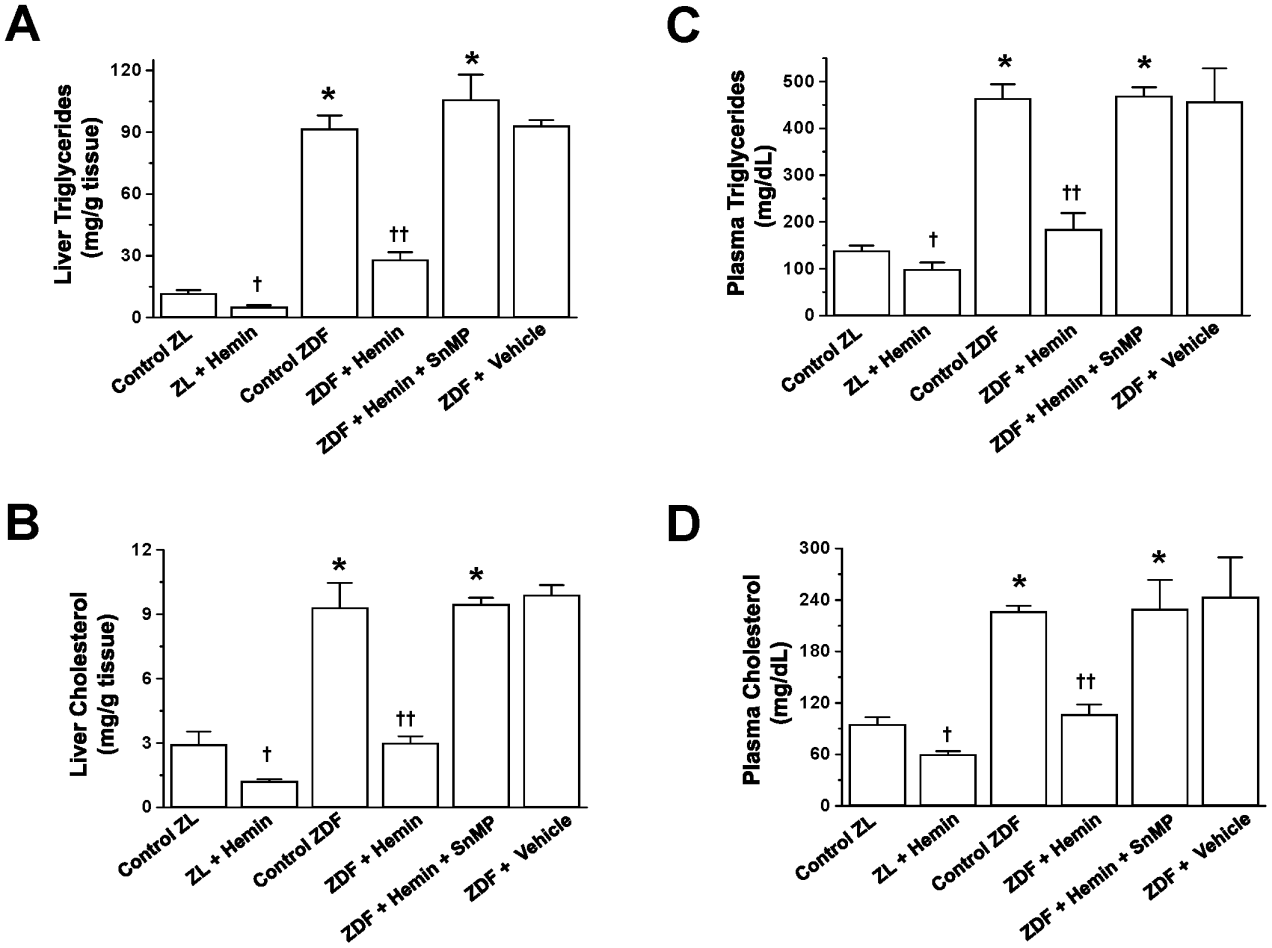
Effects of hemin, the HO inducer and SnMP, the HO inhibitor on hepatic triglycerides, hepatic cholesterol, plasma triglycerides and plasma cholesterol. Hemin therapy markedly reduced the elevated basal levels of (A) liver triglycerides, (B) liver cholesterol, (C) plasma triglycerides and (D) plasma cholesterol in ZDFs, whereas the HO blocker, SnMP nullified the effects. Hemin therapy also lowered hepatic triglycerides/cholesterol and plasma triglycerides/cholesterol in ZLs. Bars represent means ± SEM; *n = 6* rats per group (*p<0.01 *vs* Control-ZL; ^†^p<0.05, ^††^p<0.01 *vs* Control-ZL or Control-ZDF).

Since elevated hepatic triglycerides and high levels of hepatic cholesterol are common denominators of liver disease [7]–[9], we investigated the effects of hemin therapy on hepatic triglycerides and hepatic cholesterol. In ZDF-controls, the basal levels of liver triglycerides and liver cholesterol were significantly elevated as compared to ZL-controls (**Figs. 4A and 4B**). Similarly, the basal levels of plasma triglycerides and plasma cholesterol in ZDF-controls were markedly elevated (**Figs. 4C and 4D**). Interestingly, treatment with hemin greatly reduced the elevated basal levels of liver triglycerides/cholesterol and plasma triglycerides/cholesterol (**Fig. 4**). Hemin therapy also reduced triglycerides and cholesterol in ZL-controls, though to a lesser extent as compared to ZDFs.

### Hemin Therapy Abated the Expression of Markers for the Pro-inflammatory M1-macrophage, While Enhancing Markers for the Anti-inflammatory M2-phenotype in Hepatic Tissue

**Figure 5:**
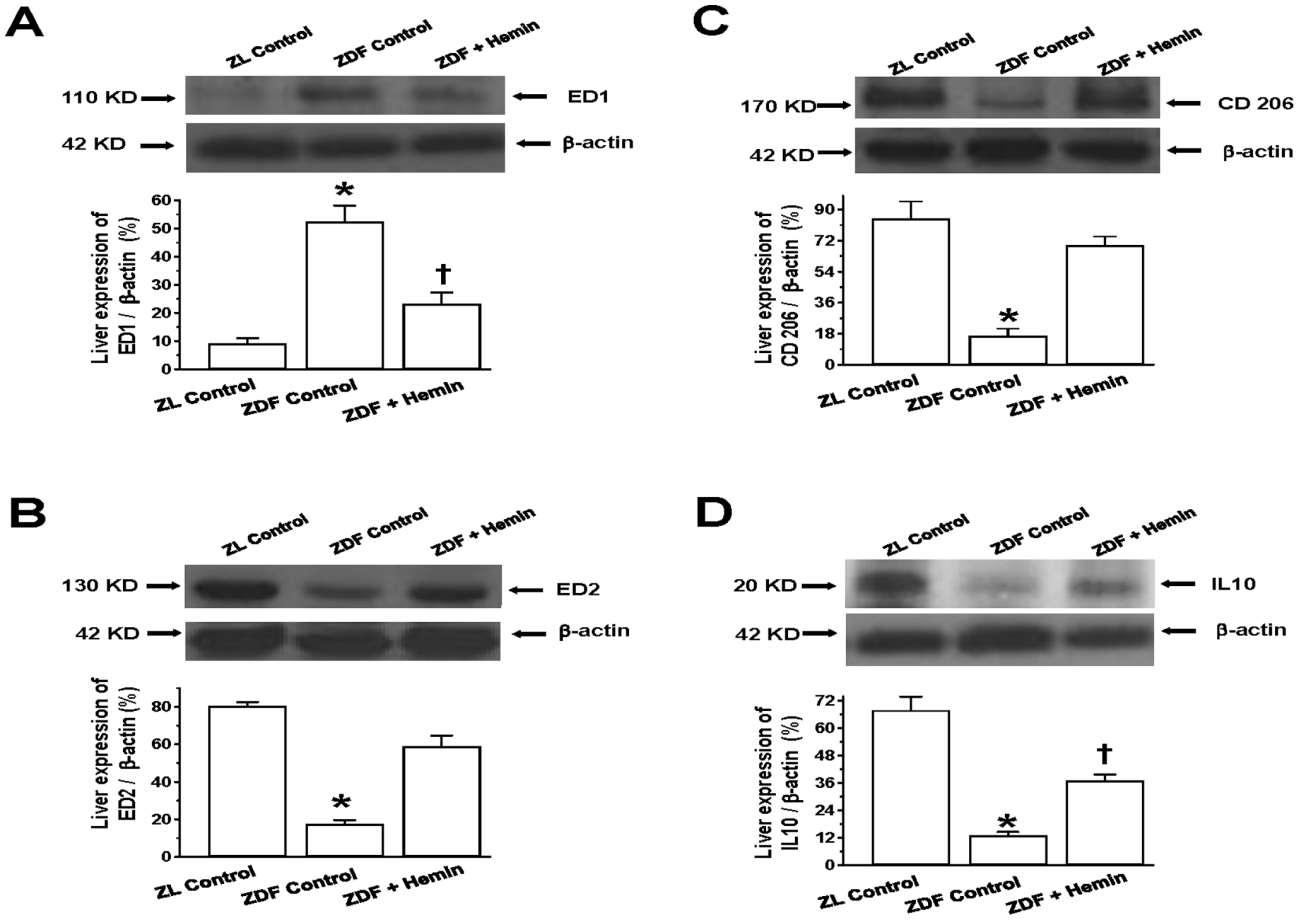
Effects of hemin on hepatic expression of ED1, ED2, CD206 and IL10. Representative Western immunoblot and relative densitometric analyses of expressed protein normalized with β-actin indicates that hemin therapy suppressed the elevated basal expression of (A) the pro-inflammatory macrophage M1-phenotype marker, ED, but enhanced the expression of several anti-inflammatory macrophage M2-phenotye markers such as (B) ED2, (C) CD206 and (D) IL10 in ZDFs. Bars represent means ± SEM; *n = 4* rats per group (*p<0.01 *vs* Control-ZL, ^†^p<0.05 *vs* Control-ZL).

Given that macrophage infiltration is implicated in hepatic impairment [50]–[52], we used specific markers namely, ED1 to quantify the pro-inflammatory M1-phenotype, and ED2, CD206 and IL10 for the assessment of anti-inflammatory M2-phenotype [19], [53], [54]. Moreover IL10 is protective against fatty liver disease [18]. Our Western immunoblotting and relative densitometry revealed that the basal expression of ED1 in the liver of ZDF-controls was significantly elevated as compared to the ZL-controls (**Fig. 5A**). Interestingly hemin therapy significantly attenuated the elevated expression of the pro-inflammatory M1-phenotype marker, ED1 (**Fig. 5A**).

To further investigate the effects of hemin on inflammation, we also determined the expression of the M2-phenotype. Our results indicate that the basal expression levels of different anti-inflammatory M2-phenotype markers including ED2, CD206 and IL10 were markedly reduced in ZDF-controls as compared to ZL-controls (**Figs. 5B, 5C and 5D**). Interestingly, hemin therapy robustly enhanced the depressed basal expression levels of ED2 (**Fig. 5B**), CD206 (**Fig. 5C**) and IL10 (**Fig. 5D**), suggesting that hemin therapy may selectively modulate the polarization of macrophage toward the M2-phenotype that dampens inflammation.

### Hemin Therapy Suppressed Macrophage Infiltration in the Liver

**Figure 6:**
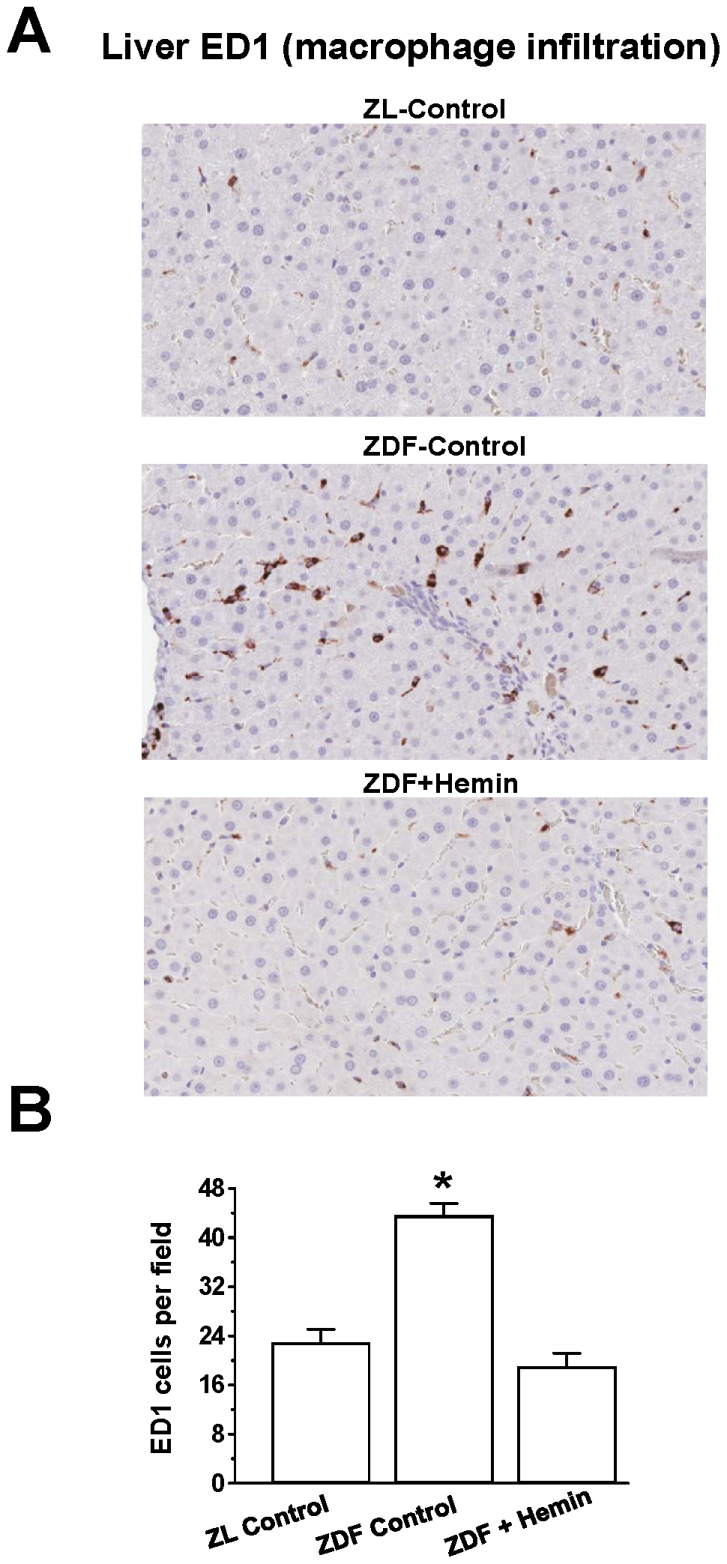
Effect of hemin therapy on macrophage infiltration in the liver. (A) Representative image indicates that macrophage infiltration (ED1-positive cells stained dark brown) in liver sections were elevated in ZDF-controls as compared to ZL-controls, but were reduced in hemi-treated ZDFs. (Magnification×200). (B) Quantitative analyses per field revealed that in ZDF-controls, macrophage infiltration was markedly elevated as compared to ZL-control, but was significantly reduced in hemi-treated ZDFs. Bars represent means ± SEM; *n = 4–6* rats per group (*p<0.01 *vs* all groups).

Since our Western immunoblotting data indicated that hemin therapy abated the expression of ED1 a marker of the pro-inflammatory macrophage M1-phenotype (**Fig. 5A**), we did immunohistochemistry to assess macrophage infiltration in hepatic tissue (**Fig. 6A**). Images of hepatic sections from ZL-controls were almost devoid of cells with the characteristic dark-brown ED1-positive staining. In contrast, liver sections from ZDF-controls showed marked increase of ED1-positive dark-brown cells as compared to ZL-controls, suggesting increased macrophage infiltration in ZDFs (**Fig. 6A**). Interestingly, hemin therapy greatly attenuated the number of dark-brown stained macrophages, suggesting reduction of macrophage infiltration in hemin-treated ZDFs. These observations were further confirmed by quantitative ED1 scoring (**Fig. 6B**), which showed a significant reduction of ED1-positive cells in hemin-treated ZDFs as compared to ZDF-controls.

### Hemin Reduced the Expression of Profibrotic and Extracellular Matrix Proteins in the Liver

**Figure 7:**
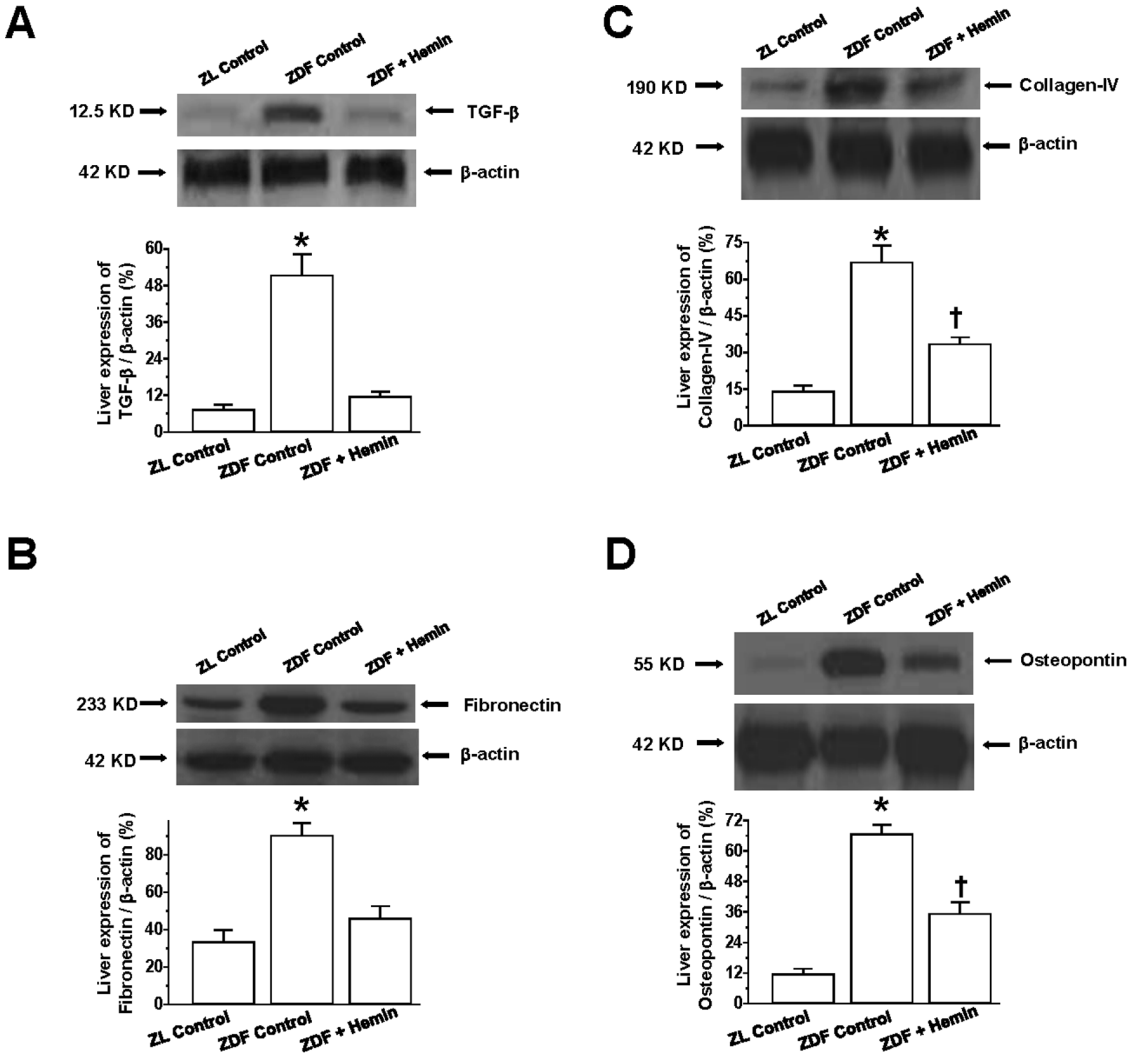
Effect of hemin therapy on liver expression of TGF-β, fibronectin, collagen-IV and osteopontin in ZDFs. Representative Western immunoblots and relative densitometry revealed that hemin therapy markedly reduced the elevated basal expression of (A) TGF-β, (B) fibronectin, (C) collagen-IV and (D) osteopontin in ZDFs. Bars represent means ± SEM; *n = 4* rats per group (*p<0.01 *vs* Control-ZL; ^†^p<0.05 *vs* Control-ZL).

Since elevated deposition of extracellular matrix is implicated in liver damage [32]–[36], we measured the levels of profibrotic and extracellular matrix proteins such as osteopontin, collagen, fibronectin and TGF-β in the liver. Moreover, TGF-β is known to activate hepatic fat to accentuate the deposition of extracellular matrix proteins [34]. In ZDF-controls the basal expression of TGF-β was significantly elevated as compared to ZL-controls (**Fig. 7A**), but was greatly attenuated by hemin.

Since TGF-β mobilizes the extracellular the matrix by stimulating fibronectin and collagen to cause fibrosis [55], so we also measured the expressions of fibronectin and collagen-IV. In ZDF-controls, the basal expressions of fibronectin and collagen-IV were significantly elevated as compared to ZL-controls, but were attenuated by hemin therapy (**Figs. 7B and 7C**). It is noteworthy that hemin effectively restored fibronectin to comparable levels as in the ZL-control, but collagen-IV was not reinstated to the levels of ZL-controls, suggesting that hemin may have greater selectivity against fibronectin. The reason for the selective effect is unknown.

We also assessed the expression of osteopontin because osteopontin is implicated in hepatic fibrosis and injury [32]. In ZDF-controls, the basal expression of osteopontin was markedly elevated as compared to ZL-controls, but was significantly reduced by hemin therapy, although similar levels as observed in the ZL-controls were not reinstated (**Fig. 7D**).

### Hemin Therapy Suppressed Hepatic Histo-pathological Lesions

**Figure 8:**
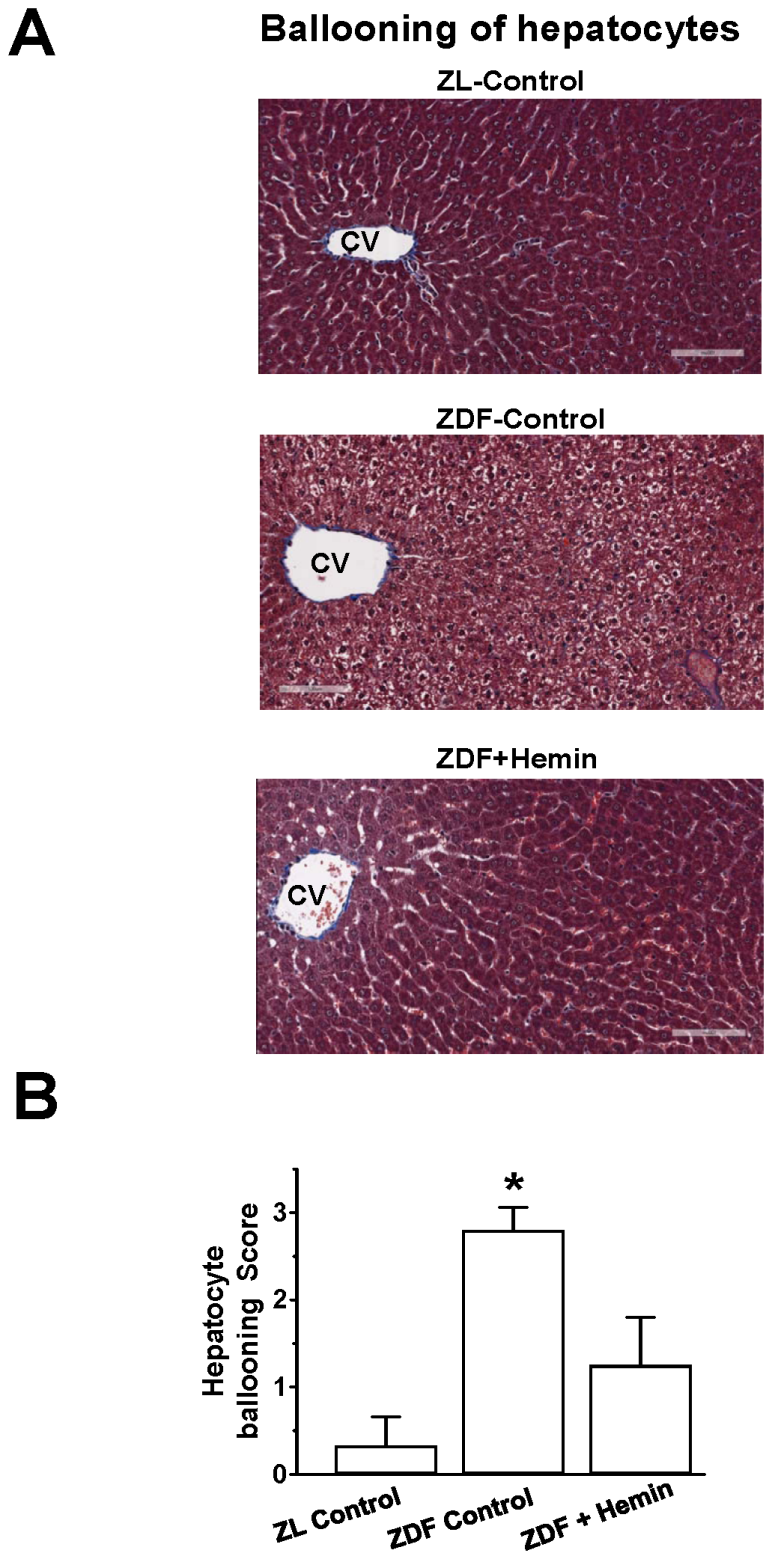
Effect of hemin therapy on hepatic histo-pathological lesions. (A) Representative image after Masson’s trichrome staining revealed severe hepatocyte ballooning injury with inflammatory cell infiltration around the central vein (CV) region in ZDFs, which interestingly were attenuated in hemin-treated ZDF (Magnification×200). (B) Semi-quantitative evaluation showed that hemin therapy significantly reduced hepatocyte ballooning injury in ZDFs. Bars represent means ± SEM; *n = 4–6* rats per group (*p<0.01 *vs* all groups).

Hepatocyte ballooning injury and fibrosis are common histo-physiological denominators in fatty-liver diseases such as NAFLD [1], [5], [32], [34]–[36]. To further investigate the effects of hemin therapy on liver lesions, we did histological and morphometric analyses using Masson’s trichrome staining. Our results indicate that sections from liver tissue obtained from ZL-controls had normal appearance and the central vein region was without hepatocyte ballooning injury (**Fig. 8A**). However, in ZDF-controls, there was greater hepatocyte ballooning injury with inflammatory cell infiltration around the central vein region, which interestingly were greatly attenuated in hemin-treated ZDF (**Fig. 8A**). These observations were further confirmed by quantitative ballooning scoring (**Fig. 8B**), which showed a significant reduction of hepatocyte ballooning injury in hemin-treated ZDFs as compared to untreated ZDF-controls.

**Figure 9:**
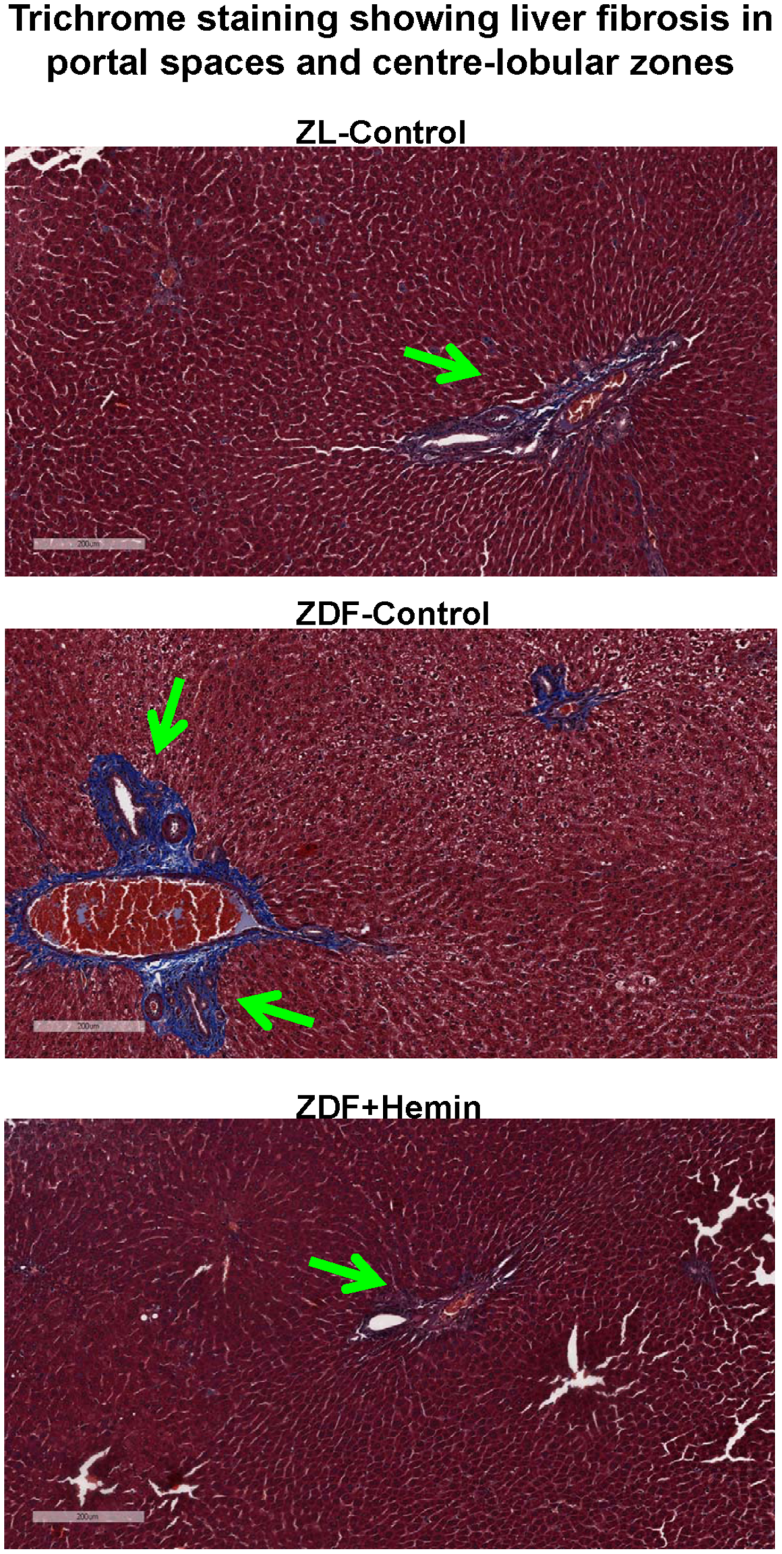
Effect of hemin therapy on hepatic fibrosis, determined by Masson” trichrome staining. (A) Representative image following Masson’s trichrome staining revealed greater fibrotic activity (blue coloration), particularly around the portal spaces with some extension toward center-lobular zones in sections from ZDFs, but interestingly were reduced in hemin-treated ZDF (Magnification×200).

To further investigate the effects of hemin therapy on hepatic morphology, fibrosis was assessed by Masson’s trichrome staining. Our results show that images of liver sections from the ZL-controls appeared relatively healthy with little Mason’s trichrome staining (blue coloration), suggesting little fibrosis (**Fig. 9**). However, in ZDF-controls, greater fibrotic activity was observed, particularly around the portal spaces with some extension towards center-lobular zones. Interestingly, hemin therapy greatly reduced the fibrotic activity in ZDF (**Fig. 9**).

## Discussion

The present study unveils several novel and/or important insights on the heptoprotective effects of hemin therapy in the condition of obesity and type-2 diabetes including; **(i)** the hemin-dependent suppression of hepatic levels of chemokines and cytokines such as MCP-1, MIP-1α, TNF-α, IL-6 and IL-1β implicated in hepatic injury; (**ii**) the reduction of plasma and hepatic triglycerides/cholesterol; **(iii)** the abrogation of extracellular matrix/profibrotic proteins in hepatic tissue including osteopontin, TGF-β, fibronectin and collagen; and **(iv)** the attenuation of hepatocyte ballooning injury to preserve hepatic morphology and function in ZDFs. Impaired hepatic function is a common phenomenon in nonalcoholic fatty liver disease [56], and with the rising prevalence and incidence of nonalcoholic fatty liver disease following the dramatic escalation of obesity, insulin resistance and overt diabetes worldwide [57], novel pharmacological agents capable of preserving hepatic function are needed. Accordingly, this study demonstrates that upregulating the HO-system with hemin abates inflammation, suppress hepatic adiposity and attenuate hepatocyte ballooning injury in ZDF, a model characterized by obesity, aberrant hepatic response to insulin, type-2 diabetes and impaired hepatic lipid metabolism [30], [31]. Hepatic adiposity and inflammation are the hallmarks of non-alcoholic fatty liver disease [9], [56], [58]. Therefore, the elevated basal levels of hepatic triglycerides, hepatic cholesterol, macrophage infiltration, pro-inflammatory cytokines/chemokines including, TNF-α, IL-6, IL-1β, MIP-1α and MCP-1 are among the multifaceted pathophysiological factors that accentuate inflammation during the progressive deterioration of hepatic morphology and function in ZDFs.

In the present study, a signature of the ailing hepatic morphology in ZDFs was evidenced the severe hepatocyte ballooning injury. However, the administration of hemin therapy to ZDFs abated macrophage infiltration and pro-inflammatory mediators and improved hepatic morphology, whereas treatment with the HO-inhibitor, SnMP accentuated the levels of inflammatory cytokines and chemokines. Importantly, the hemin-induced selective enhancement the anti-inflammatory macrophage-M2-phenotype with parallel reduction of pro-inflammatory macrophage-M1-phenotype observed in our study is an alternative anti-inflammatory mechanism by which the HO-system protects hepatic tissue. Although one study had previously reported the effects of HO-1 promoter in macrophage polarization [59], the actual expression levels of M1 and M2 macrophage phenotypes were not measured, so our study provides more solid evidence on the role of the HO system on M1 and M2 during macrophage polarization. However, the present study may be just the tip of an iceberg and further investigations are needed to fully characterize the effects of upregulating the HO system with hemin on macrophage polarization in ZDF.

Besides its effects against inflammation, hemin also suppressed osteopontin, an extracellular matrix proteins is implicated in hepatic fibrosis and injury [32]. Similarly, other extracellular matrix/profibrotic proteins such as TFG-β, collagen and fibronectin that impair liver function [32]–[36], were also reduced in hemi-treated ZDFs, with corresponding reduction of hepatic histo-pathological lesions like hepatocyte ballooning injury and fibrosis. Hemin therapy also potentiated the HO-system and cGMP in ZL-control rats and abated MIP-1α, MCP-1, TNF-α, IL-6 and IL-1β although the effect of hemin was less-intense in ZLs as compared to ZDFs with aberrant HO-system. Although the reasons for this selective effect of hemin remain unclear, it is possible that the HO-system in healthy ZLs is more stable than in unhealthy ZDFs with depressed HO-activity. Nevertheless, future studies should be designed to investigate this observation.

The emerging cytoprotective role of the HO system in nonalcoholic fatty liver disease has been well-acknowledged [60], [61]. Accordingly, the HO system has been shown to alleviate hepatic steatosis and necroinflammation in a mice model of experimental nutritional steatohepatitis [60]. Similarly, the anti-oxidant effect of the HO system was amongst the mechanisms conferred protection against nonalcoholic fatty liver disease in mice and humans [61], [62]. Therefore, the present study is a further testimony of the important hepatoprotective role of the HO system.

Collectively, our study indicates that hemin therapy restore hepatic morphology by abating hepatic adiposity, suppressing liver macrophage infiltration, inflammation, fibrosis and extracellular/profibrotic proteins implicated in hepatic lesions. The selective enhancement of anti-inflammatory macrophage-M2-phenotype with parallel reduction of pro-inflammatory macrophage-M1-phenotype and related chemokines/cytokines like TNF-α, IL-6, IL-1β, MIP-1α and MCP-1 are among the multifaceted mechanisms by which hemin restore hepatic morphology.
